# Post-Esophagectomy Dumping Syndrome: Assessing Quality of Life of Long-Term Survivors

**DOI:** 10.3390/jcm14103587

**Published:** 2025-05-21

**Authors:** Dionysios Dellaportas, Ioannis Margaris, Eleftherios Tsalavoutas, Zoi Gkiafi, Anastasia Pikouli, Despoina Myoteri, Nikolaos Pararas, Panagis M Lykoudis, Constantinos Nastos, Emmanuel Pikoulis

**Affiliations:** 1 3rd Department of Surgery, Attikon University Hospital, 12462 Athens, Greece; dellapdio@med.uoa.gr (D.D.); pikoulianastasia@gmail.com (A.P.); npararas@gmail.com (N.P.); kosnastos@yahoo.gr (C.N.); mpikoul@med.uoa.gr (E.P.); 2 4th Department of Surgery, Attikon University Hospital, 12462 Athens, Greece; tsaladive@gmail.com (E.T.); zwigiafi@gmail.com (Z.G.); p.lykoudis@ucl.ac.uk (P.M.L.); 3 Department of Pathology, Aretaieion University Hospital, 11528 Athens, Greece; dmyoteri@med.uoa.gr

**Keywords:** esophagectomy, dumping syndrome, quality of life

## Abstract

**Background/Objectives:** Survival rates for esophageal cancer patients have markedly improved. Inevitably, attention has been drawn to functional and quality-of-life problems. The aim of the current study was to investigate the prevalence of dumping syndrome in patients following esophageal resection and its correlation with postoperative quality of life. **Methods**: This cross-sectional study involved disease-free patients who underwent a potentially curative resection for esophageal or gastroesophageal junction carcinoma between January 2019 and January 2024 in a single academic institution. Patients were asked to fill in two questionnaires: the Dumping Syndrome Rating Scale (DSRS) and the QLQ-OG25. A Composite Dumping Syndrome Index (CDSI) was calculated by adding the summary severity and frequency scores for each patient. **Results:** During the study period, 42 patients underwent esophagectomy for malignant esophageal or junctional tumors. In total, 14 eligible patients responded to the questionnaires at a mean time of 19.7 (±20.8) months following their operation. Three patients (21%) reported having at least quite severe problems related to at least two dumping symptoms. Six patients (43%) reported that they avoid certain foods in order to alleviate related problems. A high CDSI score was associated with significantly increased OG25 scores for dysphagia, eating restriction, odynophagia, pain and discomfort, and reflux (*p* < 0.05). **Conclusions**: Early dumping syndrome can occur in a significant proportion of patients following esophagectomy and may adversely affect quality of life.

## 1. Introduction

The burden of esophageal cancer has been steadily rising in recent years. It is now the eighth most common cause of cancer and remains the sixth cause of cancer deaths globally [1]. Standard treatment for esophageal malignancy includes radical surgery coupled with perioperative chemotherapy or chemoradiotherapy [2]. Recent developments in surgical and oncological treatment have improved the prognosis and survivorship of esophageal cancer patients, with 40% of them being alive and disease-free long after surgery [3].

Inevitably, attention has been given to the alimentary function of patients surviving such a major operation, and the implications on their quality of life. Most patients will experience some form of gastrointestinal discomfort, weight loss, and malnutrition [4]. Some of them will also suffer from functional conditions, including dysphagia, delayed gastric conduit emptying, reflux, and dumping syndrome [5].

Dumping syndrome was first described by Hertz in 1913 and affects patients that have undergone surgery of the upper gastrointestinal tract [6]. Although it has been commonly reported after gastrectomy or bariatric operations, it may also occur following esophageal resections [5,7,8,9]. Dumping syndrome can be classified into early and late dumping. The former usually occurs 10–30 min after a meal and has both gastrointestinal and vasomotor symptoms. On the other hand, the hallmark of late dumping syndrome is hypoglycemia occurring 1–3 h after a meal [10].

Dumping syndrome in the setting of esophageal cancer surgery constitutes a poorly understood and inconsistently reported complication. According to a recent systematic review, the prevalence of dumping syndrome following esophagectomy ranges from 0 to 78%, possibly reflecting heterogeneity in definitions and diagnostic methods used among authors [11]. Furthermore, most studies have not utilized standardized clinical assessment tools, which may have resulted in severe underestimations of its occurrence.

The aim of the current study was to monitor the prevalence of post-esophagectomy early dumping syndrome and to assess its relationship with health-related quality of life in patients surviving this aggressive malignancy and extensive operation using standardized symptom-based questionnaires.

## 2. Materials and Methods

### 2.1. Study Design, Patients, and Outcomes Measured

This cross-sectional study involved patients who underwent a potentially curative resection for esophageal or gastroesophageal junction carcinoma between January 2019 and January 2024 in a single academic institution. Patients were included in the study as long as they were disease-free during their last clinical visit, which took place between April and June 2024. Eligible patients willing to participate in the study were asked to fill in two questionnaires, either in the setting of their outpatient visit or via an e-mail. All patients were operated on by the same surgical team. In all cases, an open surgical approach was performed, with a tubularized gastric conduit fashioned and placed in the posterior mediastinum. An esophagogastrostomy was performed either in the cervical region or in the thoracic cavity. No pyloric drainage procedures were employed. Patients were routinely discharged with dietary recommendations of eating small and frequent meals and avoiding drinking large amounts of fluids in relation to meals. The postoperative follow-up schedule included 3–6-month outpatient visits for the first two years postoperatively and yearly thereafter. Local or distant recurrence was suspected on clinical grounds, however diagnosed endoscopically or radiologically. The study was conducted in accordance with Helsinki’s declarations for ethics in medical research and received institutional review board approval. The primary study outcome was establishing the prevalence of dumping syndrome in patients following esophageal resection. The secondary outcome was determining the correlation between dumping syndrome and quality-of-life parameters.

### 2.2. Methodology and Definitions

Data regarding patients’ baseline characteristics, operative details, and postoperative outcomes were derived from prospectively collected electronic institutional databases. In order to measure the severity and frequency of early dumping symptoms, the validated Dumping Syndrome Rating Scale (DSRS) questionnaire was used [12]. DSRS incorporates nine cardinal dumping-related symptoms, including fatigue, palpitations, sweating/flushing, cold sweats, the need to lie down, diarrhea, nausea and vomiting, stomach cramps, and fainting esteem occurring early after the consumption of a meal. Severity is scored on a 7-point Likert scale, while frequency is scored on a 6-point Likert scale. Additional questions address symptoms occurring during the ingestion of fluids in relation to a meal, symptoms occurring during the consumption of heavily sweetened drinks, and the potential avoidance of certain foods to alleviate problems and discomfort.

Summary severity and summary frequency scales were computed by summing the severity and frequency items, respectively. We calculated the Composite Dumping Syndrome Index (CDSI) adopted by Klevebro et al. by adding the summary severity score and the summary frequency score for each patient [13]. Furthermore, based on the severity of symptoms, patients experiencing at least quite severe problems in relation to at least two dumping-related symptoms were considered likely to suffer from dumping syndrome.

In terms of health-related quality of life, the European Organization for Research and Treatment of Cancer (EORTC) QLQ-OG25 questionnaire was completed, which is validated for use in patients with esophageal and esophagogastric carcinomas [14]. The questionnaire includes 25 questions in total. Six multi-item scales are used for dysphagia, eating restriction, reflux, odynophagia, pain and discomfort, and anxiety. Ten single-item questions address additional quality-of-life parameters. Responses to the questions were scored and linearly transformed according to the EORTC QLQ-OG25 scoring manual. A higher score, in any of the scales or items included, indicates worse symptomatology.

### 2.3. Statistical Analysis

Continuous variables are presented as means (±SD) or medians (IQR), according to their data distribution. The normality of distribution was assessed using the one-sample Kolmogorov–Smirnov test. Categorical variables are presented as counts with valid percentages. For between-group comparisons, the Mann–Whitney U test or the independent samples *t*-test was used. For correlations between continuous variables, Spearman’s rank correlation was used. Statistical analysis was performed using IBM SPSS Statistics for Windows, version 19.0 (IBM Corp, Armonk, NY, USA).

## 3. Results

During the study period, 42 patients underwent esophagectomy for malignant esophageal or junctional tumors. At the time of assessment, 25 patients were excluded due to recurrence or death. Out of 17 eligible patients, 14 patients responded to the questionnaires and were included in the analysis. The response rate was 82%. The mean time elapsed from the date of operation was 19.7 (±20.8) months.

The demographic and baseline characteristics of the study population are presented in Table 1. All patients, except for one, were male. The mean age of the patients at the time of the operation was 63.07 (±10.38) years. Average BMI was 22.79 (±3) kg/m^2^. Most of the patients were operated for an adenocarcinoma of the lower esophagus or the gastroesophageal junction. The most commonly performed procedure was an open left thoracoabdominal approach. Six patients (43%) experienced a deviation from the normal postoperative course, most commonly due to respiratory complications. During the course of the follow-up, two patients received a diagnostic upper gastrointestinal endoscopy for persistent complaints of nausea and vomiting. No evident cause, such as anastomotic stricture or delayed gastric conduit emptying, could be identified upon endoscopic evaluation. None of the patients included in the study received a pharmacological or endoscopic intervention related to their symptoms.

The DSRS scores are presented in detail in Table 2. The median (IQR) values for the summary severity and the summary frequency scales were 17 (13.75–23) and 12.50 (10–14), respectively. Reportedly, the most severe dumping-related symptoms in our cohort of patients were as follows: (a) the need to lie down after the consumption of a meal, and (b) pain, vomiting, or the need to stop when drinking fluids in relation to a meal. Taking into consideration the severity as well as the frequency of symptoms (Total Index), the need to lie down following the ingestion of a meal carried the greatest burden, with a median (IQR) value of 3 (1–18.5). Four patients (29%) reported absent or only mild inconvenience in relation to the dumping-related symptoms. On the other hand, three patients reported having at least quite severe problems related to at least two dumping symptoms. Hence, these three patients (21% of the study population) were considered likely to suffer from dumping syndrome. Six patients (43%) reported that they avoid certain foods in order to alleviate problems caused by food consumption. Foods commonly avoided included whole meat and high-fiber foods.

The median (IQR) scores derived from the QLQ-OG25 scales and items are listed in Table 3. The most frequently reported symptoms were early satiety, followed by trouble enjoying meals, taking too long to complete a meal, thinking about illness, and worrying about health in the future. The OG25 scores for dysphagia (z = −2.63; *p* = 0.01), eating restriction (z = −2.21; *p* = 0.03), odynophagia (z = −2.69; *p* = 0.01), and pain and discomfort (z = −2.76; *p* = 0.01) were significantly higher for patients likely suffering from dumping syndrome. The median (IQR) Composite Dumping Syndrome Index (CDSI) score was 29 (25.3–36.8). Furthermore, increased CDSI scores were associated with significantly increased OG25 scores for dysphagia (r (12) = 0.54; *p* = 0.05), eating restriction (r (12) = 0.61; *p* = 0.02), odynophagia (r (12) = 0.61; *p* = 0.02), pain and discomfort (r (12) = 0.70; *p* = 0.01), and reflux (r (12) = 0.70; *p* = 0.01) (Figures S1–S5). No statistically significant correlation was found between age, sex, BMI, time elapsed from the operation, reconstruction technique, and CDSI.

## 4. Discussion

This original cross-sectional study was designed to evaluate the prevalence of post-esophagectomy early dumping syndrome and its correlation with quality-of-life parameters using disease-specific symptom-based questionnaires. One in five patients reported a cluster of symptoms severe enough to indicate that they have been suffering from dumping syndrome postoperatively. Forty-three percent of the patients reported avoiding certain foods to reduce issues associated with food consumption. Furthermore, dumping syndrome and a high burden of related symptoms had a significantly negative impact on health-related quality-of-life aspects, including dysphagia, eating restrictions, and pain and discomfort.

Breakthroughs in medical research and the advent of multimodality treatment have significantly improved survival rates for esophageal cancer patients [3]. At the same time, almost 70% of patients are affected by symptoms related to their esophagectomy more than a year after the operation, including fatigue, early satiety, and reflux [15]. Dumping syndrome is a notable complication of gastric and bariatric surgery, but variably reported in the context of esophagectomy. A recent systematic review and meta-analysis investigating the occurrence of dumping syndrome following curative intent surgery in esophageal cancer patients included 16 studies and reported a weighed prevalence of 27%, with a high heterogeneity among studies. A subgroup analysis evaluating only the three studies that used specialized questionnaires found a strikingly high pooled prevalence of 67%, with reduced heterogeneity [16].

According to our results, the most common severe symptoms were the need to lie down after the consumption of a meal and the experience of pain, vomiting, or the need to stop when drinking fluids in relation to a meal (each one reported by 21% of the patients). Other symptoms commonly reported in the literature are diarrhea, abdominal cramps or discomfort, early satiety, and nausea [17,18].

The current cohort of patients was operated on by a single surgical team and with a standardized technique. In all cases, a tubularized gastric conduit was utilized and no pyloric drainage procedures were employed. Even though evidence in the literature is contradictory, pyloric drainage has been linked to higher rates of postoperative dumping syndrome and debilitating bile reflux esophagitis, while adding complexity and time to the operation [19,20]. Hence, it has been suggested that, instead of performing routine pyloromyotomy or pyloroplasty, placing a narrow-gauged gastric tube high in the cervical or the thoracic region can prevent postoperative delayed gastric emptying, reduce the incidence of dumping syndrome, and enhance quality of life [13]. Additionally, if needed, a postoperative endoscopic intervention, usually in the form of pyloric balloon dilatation, can effectively manage persistent gastric outlet obstructive symptoms [21,22]. Klevebro et al. reported no association between the type of open surgical approach and a compound dumping symptom score [13]. In a propensity-score-matched analysis by Mehran et al., dumping symptoms were equally observed among patients treated with a minimally invasive and an open approach [18]. However, in a recent cohort study involving 188 patients, open esophagectomy was related to more severe dumping symptoms [23]. It has also been suggested by other reports that an open approach may lead to worse functional outcomes and health-related quality-of-life parameters [24,25].

In our study, a high CDSI score was related to worse dysphagia, odynophagia, pain and discomfort, and even reflux scores. Intriguingly, finding a correlation between these distinct functional symptoms is challenging. Dysphagia is commonly attributed to an anastomotic stricture, due to local ischemia and tension, or to long-term reflux-induced stenosis, due to the anatomic absence of a lower esophageal sphincter. On the other hand, the impairment of gastric storage and accommodation capacity, along with vagal denervation, can potentiate rapid emptying towards the small bowel and give rise to dumping symptoms [5]. Despite the uncertainties as to the different pathophysiologic mechanisms involved, it seems that patients undergoing esophagectomy may suffer from a spectrum of symptoms and syndromes that significantly overlap [25]. In the study by Klevebro et al., a high compound dumping symptom score was associated with significantly reduced functional scores and significantly increased symptomatic scores on the EORTC QLQ-C30 questionnaire [13]. Likewise, Bennett et al. found that patients suffering from dumping syndrome exhibited significantly worse health-related quality-of-life scores postoperatively, including in relation to physical function, social function, eating restrictions, and pain and discomfort [9]. Furthermore, a strong correlation between increased dumping scores and fatigue, dyspnea, and social function was established.

According to our results, there was no association between the time elapsed from the operation and the rate of dumping syndrome. In a study by De Leyn et al., an improvement in symptoms was observed within one year of surgery [26]. Nonetheless, in a recent prospective study involving 66 patients, the prevalence of dumping syndrome significantly increased with increasing time postoperatively, albeit follow-up was also concluded at 12 months [9].

Additionally, no significant differences were noted with regard to the age of the patients or sex. However, there are reports that the syndrome may predominately affect younger patients [13,17].

No specific association was revealed between preoperative BMI and dumping syndrome, which is concordant with the results of another study reporting equivalent rates of dumping syndrome among obese and non-obese patients [27]. The relation between postoperative dumping syndrome and weight loss remains elusive. It is well known that esophageal carcinoma survivors suffer from postoperative malnutrition and even sarcopenia, both of which have been linked to poor survival outcomes [28]. It seems only reasonable in this setting that dumping syndrome may accelerate weight loss and worsen nutritional deficits. Contrary, however, to this notion, a study involving bariatric patients found no relation between dumping syndrome and concomitant weight loss [29]. Naturally, the findings cannot be readily generalized to patients with esophageal malignancies, as weight loss and malnutrition in this context are complex and multifactorial problems.

### Limitations

Despite achieving a high response rate from our pool of patients and using methodologically validated instruments, our study has some obvious weaknesses. Possible sources of bias include the small sample size, the fact that patients stem from a single institution and were operated on by a single surgical team, and the lack of a long-term follow-up. Additionally, no objective testing for dumping syndrome was used. Finally, due to the cross-sectional design of the study, comparisons to pretreatment baseline levels of quality of life and dumping symptom measures could not be made. However, in the current study, the symptom-based diagnosis of dumping syndrome was supported by the use of a specialized questionnaire, and we believe our results may be useful in promoting awareness of this functional syndrome and its quality-of-life implications among clinicians caring for patients with esophageal malignancies.

## 5. Conclusions

In summary, the above results indicate that early dumping syndrome can occur in a significant proportion of patients after esophagectomy. The current study also highlights the adverse effects that dumping-related symptoms may exhibit on health-related quality-of-life aspects. Vigilance is required to identify patients who have dumping symptoms and require further medical or nutritional support.

## Figures and Tables

**Table 1:** Demographic and baseline characteristics.

Male Sex			13 (93%)
Age; mean (±SD)			63.07 (±10.38)
Body mass index (BMI); mean (±SD)			22.79 (±3.00)
Time from operation		<1 year	5 (36%)
		1–2 years	5 (36%)
		>2 years	4 (29%)
ASA ^ǂ^ score		I	2 (14%)
		II	5 (36%)
		III	7 (50%)
		IV–V	0 (0)
Histology		Adenocarcinoma	14 (100%)
		Squamous cell carcinoma	0 (0)
Clinical tumor stage		T1	0 (0)
		T2	4 (29%)
		T3	8 (57%)
		T4	2 (14%)
Clinical nodal stage		N0	2 (14%)
		N1	7 (50%)
		N2	5 (36%)
		N3	0 (0)
Tumor location		Upper/middle esophagus	2 (14%)
		Lower esophagus/gastroesophageal junction	12 (86%)
Neoadjuvant treatment		No	2 (14%)
		Chemoradiotherapy	2 (14%)
		Chemotherapy	10 (71%)
Surgical approach		Ivor Lewis	4 (29%)
		Three-stage	2 (14%)
		Left thoracoabdominal	6 (43%)
		Transhiatal	2 (14%)
Postoperative complications		None	8 (57%)
		CD ^‡^ I	0 (0)
		CD II	3 (21%)
		CD IIIa	1 (7%)
		CD IIIb	2 (14%)
		CD IV–V	0 (0)

^ǂ^ ASA: American Society of Anesthesiologists physical status score. ^‡^ CD: Clavien–Dindo classification.

**Table 2:** DSRS scores.

Severity Scale *	**1**	**2**	**3**	**4**	**5**	**6**	**7**
Fatigue	7 (50%)	3 (21%)	2 (14%)	0 (0)	2 (14%)	0 (0)	0 (0)
Palpitations	10 (71%)	2 (14%)	0 (0)	1 (7%)	1 (7%)	0 (0)	0 (0)
Sweating/flushing	9 (64%)	4 (29%)	0 (0)	0 (0)	0 (0)	0 (0)	1 (7%)
Cold sweats	13 (93%)	0 (0)	1 (7%)	0 (0)	0 (0)	0 (0)	0 (0)
Need to lie down	6 (43%)	3 (21%)	0 (0)	2 (14%)	1 (7%)	1 (7%)	1 (7%)
Diarrhea	9 (64%)	4 (29%)	0 (0)	0 (0)	1 (7%)	0 (0)	0 (0)
Nausea and vomiting	8 (57%)	3 (21%)	0 (0)	1 (7%)	2 (14%)	0 (0)	0 (0)
Stomach cramps	10 (71%)	2 (14%)	0 (0)	2 (14%)	0 (0)	0 (0)	0 (0)
Fainting esteem	13 (93%)	0 (0)	0 (0)	1 (7%)	0 (0)	0 (0)	0 (0)
Pain/vomiting/“stop” with fluids	6 (43%)	4 (29%)	0 (0)	1 (7%)	2 (14%)	1 (7%)	0 (0)
Symptoms with heavily sweetened drinks	11 (79%)	1 (7%)	1 (7%)	1 (7%)	0 (0)	0 (0)	0 (0)
Frequency Scale **	**1**	**2**	**3**	**4**	**5**	**6**	
Fatigue	10 (71%)	3 (21%)	1 (7%)	0 (0)	0 (0)	0 (0)	
Palpitations	12 (86%)	1 (7%)	1 (7%)	0 (0)	0 (0)	0 (0)	
Sweating/flushing	11 (79%)	2 (14%)	1 (7%)	0 (0)	0 (0)	0 (0)	
Cold sweats	13 (93%)	0 (0)	1 (7%)	0 (0)	0 (0)	0 (0)	
Need to lie down	7 (50%)	2 (14%)	3 (21%)	1 (7%)	1 (7%)	0 (0)	
Diarrhea	10 (71%)	3 (21%)	1 (7%)	0 (0)	0 (0)	0 (0)	
Nausea and vomiting	9 (64%)	2 (14%)	1 (7%)	1 (7%)	0 (0)	1 (7%)	
Stomach cramps	11 (79%)	1 (7%)	1 (7%)	0 (0)	1 (7%)	0 (0)	
Fainting esteem	11 (79%)	3 (21%)	0 (0)	0 (0)	0 (0)	0 (0)	

* For severity scale: 1 = no trouble at all, 2 = minor inconvenience, 3 = mild trouble, 4 = moderate trouble, 5 = quite severe problems, 6 = severe problems, 7 = very severe problems. ** For frequency scale: 1 = no trouble at all, 2 = less than once a week, 3 = once a week, 4 = a few times per week, 5 = once per day, 6 = several times a day.

**Table 3:** Items’ median (IQR) scores on QLQ-OG25 scale.

Dysphagia	11 (0–22)
Eating	37.5 (23–46)
Reflux	33 (0–37.25)
Odynophagia	17 (0–37.25)
Pain and discomfort	8.5 (0–41.5)
Anxiety	50 (24.75–83)
Eating with others	0 (0–0)
Dry mouth	0 (0–8.25)
Trouble with taste	0 (0–33)
Body image	0 (0–33)
Trouble swallowing saliva	0 (0–0)
Choked when swallowing	0 (0–0)
Trouble with coughing	0 (0–8.25)
Trouble talking	0 (0–8.25)
Weight loss	16.5 (0–33)
Hair loss	0 (0–16.75)

## Data Availability

Dataset available on request from the authors.

## Supplementary Materials

The following supporting information can be downloaded at: https://www.mdpi.com/article/10.3390/jcm14103587/s1: Figure S1: Scatter plot of Composite Dumping Syndrome Index (CDSI) score and OG25 score for dysphagia; Figure S2: Scatter plot of Composite Dumping Syndrome Index (CDSI) score and OG25 score for eating restriction; Figure S3: Scatter plot of Composite Dumping Syndrome Index (CDSI) score and OG25 score for odynophagia; Figure S4: Scatter plot of Composite Dumping Syndrome Index (CDSI) score and OG25 score for pain and discomfort; Figure S5: Scatter plot of Composite Dumping Syndrome Index (CDSI) score and OG25 score for reflux.

## Abbreviations

The following abbreviations are used in this manuscript:

DSRS	Dumping Syndrome Rating Scale
CDSI	Composite Dumping Syndrome Index
EORTC	European Organization for Research and Treatment of Cancer
BMI	Body mass index
ASA	American Society of Anesthesiologists physical status score
CD	Clavien–Dindo classification

## References

[1] Uhlenhopp D.J., Then E.O., Sunkara T., Gaduputi V. (2020). Epidemiology of esophageal cancer: Update in global trends, etiology and risk factors. Clin. J. Gastroenterol..

[2] Harada K., Rogers J.E., Iwatsuki M., Yamashita K., Baba H., Ajani J.A. (2020). Recent advances in treating oesophageal cancer. F1000Research.

[3] Shapiro J., van Lanschot J.J.B., Hulshof M.C.C.M., van Hagen P., van Berge Henegouwen M.I., Wijnhoven B.P.L., van Laarhoven H.W.M., Nieuwenhuijzen G.A.P., Hospers G.A.P., Bonenkamp J.J. (2015). Neoadjuvant chemoradiotherapy plus surgery versus surgery alone for oesophageal or junctional cancer (CROSS): Long-term results of a randomised controlled trial. Lancet Oncol..

[4] Martin L., Lagergren J., Lindblad M., Rouvelas I., Lagergren P. (2007). Malnutrition after oesophageal cancer surgery in Sweden. Br. J. Surg..

[5] Ukegjini K., Vetter D., Fehr R., Dirr V., Gubler C., Gutschow C.A. (2021). Functional syndromes and symptom-orientated aftercare after esophagectomy. Langenbeck’s Arch. Surg..

[6] Hertz A.F. (1913). The Cause and Treatment of Certain Unfavorable After-effects of Gastro-enterostomy. Ann. Surg..

[7] Eagon J.C., Miedema B.W., Kelly K.A. (1992). Postgastrectomy syndromes. Surg. Clin. N. Am..

[8] Mallory G.N., Macgregor A.M., Rand C.S. (1996). The Influence of Dumping on Weight Loss After Gastric Restrictive Surgery for Morbid Obesity. Obes. Surg..

[9] Bennett S., Murphy C.F., Fanning M., Reynolds J.V., Doyle S.L., Donohoe C.L. (2022). The impact of nutrition and gastrointestinal symptoms on health-related quality of life in survivorship after oesophageal cancer surgery. Clin. Nutr. Open Sci..

[10] van Beek A.P., Emous M., Laville M., Tack J. (2017). Dumping syndrome after esophageal, gastric or bariatric surgery: Pathophysiology, diagnosis, and management. Obes. Rev..

[11] Boshier P.R., Huddy J.R., Zaninotto G., Hanna G.B. (2017). Dumping syndrome after esophagectomy: A systematic review of the literature. Dis. Esophagus.

[12] Laurenius A., Olbers T., Näslund I., Karlsson J. (2013). Dumping syndrome following gastric bypass: Validation of the dumping symptom rating scale. Obes. Surg..

[13] Klevebro F., Boshier P.R., Savva K.V., Waller A., Hage L., Ni M., Hanna G.B., Low D.E. (2021). Severe Dumping Symptoms Are Uncommon Following Transthoracic Esophagectomy but Significantly Decrease Health-Related Quality of Life in Long-Term, Disease-Free Survivors. J. Gastrointest. Surg..

[14] Lagergren P., Fayers P., Conroy T., Stein H.J., Sezer O., Hardwick R., Hammerlid E., Bottomley A., Van Cutsem E., Blazeby J.M. (2007). Clinical and psychometric validation of a questionnaire module, the EORTC QLQ-OG25, to assess health-related quality of life in patients with cancer of the oesophagus, the oesophago-gastric junction and the stomach. Eur. J. Cancer.

[15] Markar S.R., Sounderajah V., Johar A., Zaninotto G., Castoro C., Lagergren P., Elliott J.A., Gisbertz S.S., Mariette C., Alfieri R. (2021). Patient-reported outcomes after oesophagectomy in the multicentre LASER study. Br. J. Surg..

[16] Lin Y., Wang H., Qu Y., Liu Z., Lagergren P., Xie S.H. (2024). Occurrence of Dumping Syndrome After Esophageal Cancer Surgery: Systematic Review and Meta-analysis. Ann. Surg. Oncol..

[17] McLarty A.J., Deschamps C., Trastek V.F., Allen M.S., Pairolero P.C., Harmsen W.S. (1997). Esophageal resection for cancer of the esophagus: Long-term function and quality of life. Ann. Thorac. Surg..

[18] Mehran R., Rice D., El-Zein R., Huang J.L., Vaporciyan A., Goodyear A., Mehta A., Correa A., Walsh G., Roth J. (2011). Minimally invasive esophagectomy versus open esophagectomy, a symptom assessment study. Dis. Esophagus.

[19] Wang L.S., Huang M.H., Huang B.S., Chien K.Y. (1992). Gastric substitution for resectable carcinoma of the esophagus: An analysis of 368 cases. Ann. Thorac. Surg..

[20] Velanovich V. (2003). Esophagogastrectomy without pyloroplasty. Dis. Esophagus.

[21] Mertens A., Gooszen J., Fockens P., Voermans R., Gisbertz S., Bredenoord A., Henegouwen M.I.v.B. (2021). Treating Early Delayed Gastric Tube Emptying after Esophagectomy with Pneumatic Pyloric Dilation. Dig. Surg..

[22] Lanuti M., de Delva P.E., Wright C.D., Gaissert H.A., Wain J.C., Donahue D.M., Allan J.S., Mathisen D.J. (2007). Post-esophagectomy gastric outlet obstruction: Role of pyloromyotomy and management with endoscopic pyloric dilatation. Eur. J. Cardio-Thorac. Surg..

[23] Anandavadivelan P., Wikman A., Malberg K., Martin L., Rosenlund H., Rueb C., Johar A., Lagergren P. (2021). Prevalence and intensity of dumping symptoms and their association with health-related quality of life following surgery for oesophageal cancer. Clin. Nutr..

[24] Maas K.W., Cuesta M.A., van Berge Henegouwen M.I., Roig J., Bonavina L., Rosman C., Gisbertz S.S., Biere S.S.A.Y., van der Peet D.L. (2015). Quality of Life and Late Complications After Minimally Invasive Compared to Open Esophagectomy: Results of a Randomized Trial. World J. Surg..

[25] Dirr V., Vetter D., Sartoretti T., Schneider M.A., Da Canal F., Gutschow C.A. (2023). Quality of Life and Independent Factors Associated with Poor Digestive Function after Ivor Lewis Esophagectomy. Cancers.

[26] De Leyn P., Coosemans W., Lerut T. (1992). Early and late functional results in patients with intrathoracic gastric replacement after oesophagectomy for carcinoma. Eur. J. Cardio-Thorac. Surg..

[27] Scipione C.N., Chang A.C., Pickens A., Lau C.L., Orringer M.B. (2007). Transhiatal esophagectomy in the profoundly obese: Implications and experience. Ann. Thorac. Surg..

[28] Anandavadivelan P., Martin L., Djärv T., Johar A., Lagergren P. (2018). Nutrition Impact Symptoms Are Prognostic of Quality of Life and Mortality After Surgery for Oesophageal Cancer. Cancers.

[29] Banerjee A., Ding Y., Mikami D.J., Needleman B.J. (2013). The role of dumping syndrome in weight loss after gastric bypass surgery. Surg. Endosc..

