# Supplementary figures and images for: Post-Esophagectomy Dumping Syndrome: Assessing Quality of Life of Long-Term Survivors
Source: J Clin Med. 2025 May 21;14(10):3587. doi: 10.3390/jcm14103587 (PMC12112537; doi:10.3390/jcm14103587)

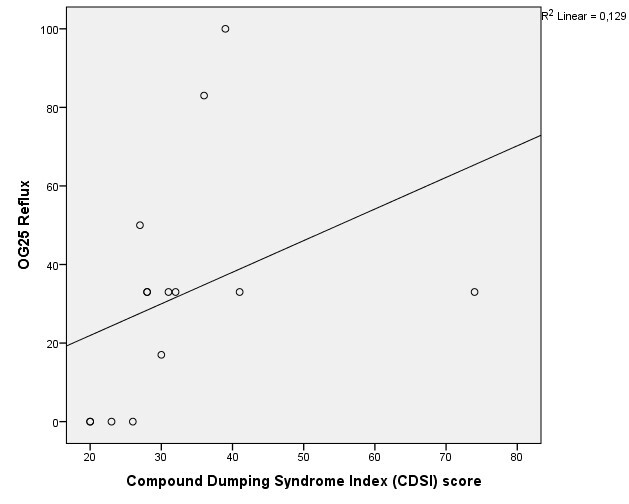

Supplement: Supplementary file 1 [file jcm-14-03587-s001.zip › Supplementary-Figure-5.png]

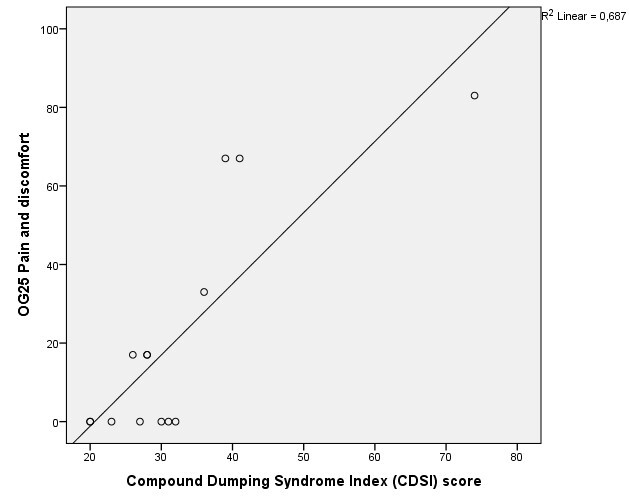

Supplement: Supplementary file 1 [file jcm-14-03587-s001.zip › Supplementary-Figure-4.png]

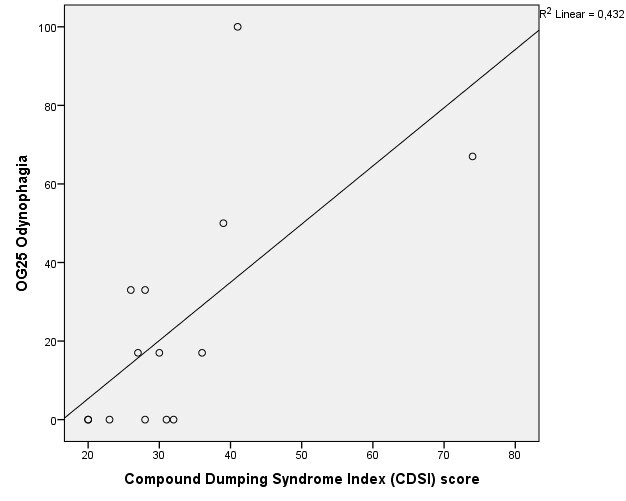

Supplement: Supplementary file 1 [file jcm-14-03587-s001.zip › Supplementary-Figure-3.png]

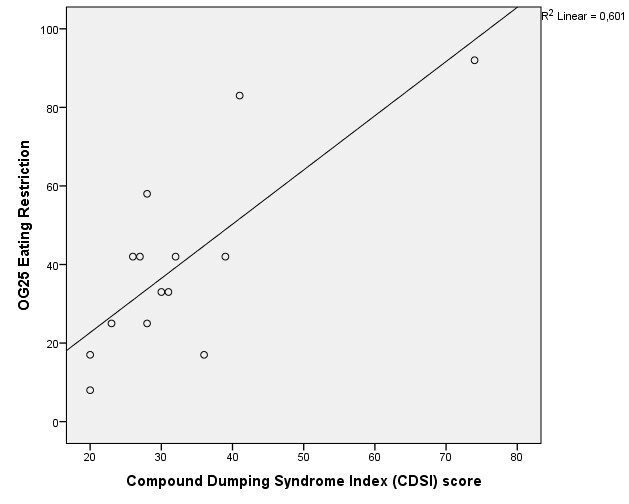

Supplement: Supplementary file 1 [file jcm-14-03587-s001.zip › Supplementary-Figure-2.png]

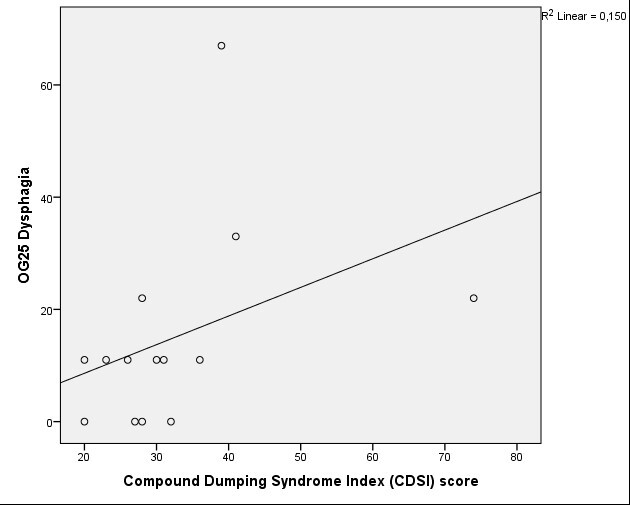

Supplement: Supplementary file 1 [file jcm-14-03587-s001.zip › Supplementary-Figure-1.png]
